# A Prospective Monitoring Study of Cytomegalovirus Infection in Non-Immunosuppressed Critical Heart Surgery Patients

**DOI:** 10.1371/journal.pone.0129447

**Published:** 2015-06-12

**Authors:** Paula Lopez Roa, Maria Jesus Perez-Granda, Patricia Munoz, Pilar Catalan, Roberto Alonso, Eduardo Sanchez-Perez, Emma Novoa, Emilio Bouza

**Affiliations:** 1 Department of Clinical Microbiology and Infectious Diseases, Hospital General Universitario Gregorio Marañón, Madrid, Spain; 2 Instituto de Investigación Biomédica Gregorio Marañón, Madrid, Spain; 3 Medicine Department, School of Medicine, Universidad Complutense, Madrid, Spain; 4 Department of Anesthesiology, Hospital General Universitario Gregorio Marañón, Madrid, Spain; University of Regensburg, GERMANY

## Abstract

**Background:**

Reactivation of cytomegalovirus (CMV) has been reported occasionally in immnunocompetent patients in the intensive care unit (ICU). The epidemiology and association of CMV infection with adverse outcome is not well defined in this population. Patients undergoing major heart surgery (MHS) are at a particularly high risk of infection. CMV infection has not been systematically monitored in MSH-ICU patients.

**Methods:**

We assessed CMV plasma viremia weekly using a quantitative polymerase chain reaction assay in a prospective cohort of immunocompetent adults admitted to the MHS-ICU for at least 72 hours between October 2012 and May 2013. Risk factors for CMV infection and its potential association with continued hospitalization or death by day 30 (composited endpoint) were assessed using univariate and multivariate logistic regression analyses.

**Results:**

CMV viremia at any level was recorded in 16.5% of patients at a median of 17 days (range, 3-54 days) after admission to the MHS-ICU. Diabetes (adjusted OR, 5.6; 95% CI, 1.8-17.4; p=0.003) and transfusion requirement (>10 units) (adjusted OR, 13.7; 95% CI, 3.9-47.8; p<0.001) were independent risk factors associated with CMV reactivation. Reactivation of CMV at any level was independently associated with the composite endpoint (adjusted OR, 12.1; 95% CI, 2.3-64; p=0.003).

**Conclusion:**

Reactivation of CMV is relatively frequent in immunocompetent patients undergoing MHS and is associated with prolonged hospitalization or death.

## Introduction

Cytomegalovirus (CMV) is a highly prevalent herpesvirus that is able to establish latency after primary infection. Reactivation from latency is classically reported in patients with solid organ transplantation, malignant hematologic disease, and AIDS [1]. In these scenarios, reactivation of CMV is associated with increased morbidity and mortality [2–7].

CMV reactivation in non-immunosupressed intensive care unit (ICU) patients is less well defined. Previous studies in this setting are mostly retrospective and show considerable variability in study design, ICU populations, and laboratory methods. As a result, the reported incidence varies from 0% to 40%, and findings on the association between CMV and adverse clinical outcomes are contradictory [8–17].

Patients undergoing major heart surgery (MHS) constitute an ICU population that is at particularly high risk of infection during the postoperative period, and incidence and related mortality are elevated [18–21]. However, data on the epidemiology, risk factors, and outcome of CMV reactivation in this setting are even scarce and out of date [22, 23]. In the present study, we prospectively assessed CMV viremia using real-time polymerase chain reaction (PCR) in a large cohort of consecutive adults admitted to a major heart surgery intensive care unit (MHS-ICU) to determine the epidemiology, risk factors, and clinical significance of CMV infection.

## Material and Methods

### Study design

Between October 2012 and May 2013, we conducted a prospective observational study in the MHS-ICU (14 beds) of Hospital Gregorio Marañon (Madrid, Spain), a 1,550-bed tertiary referral teaching institution attending a population of approximately 750,000 inhabitants. The study was approved by the local ethics committee (Comite Etico de Investigación Clínica HGU Gregorio Marañon), and written informed consent was obtained from the study participants.

New MHS-ICU admissions were screened daily by study personnel, and patients who met the inclusion criteria were enrolled and followed using standardized forms.

### Inclusion and exclusion criteria

The inclusion criteria were agreement to participate in the study, age ≥ 18 years, and admission to the MHS-ICU for at least 72 hours.

The exclusion criteria were inability to provide informed consent, age ≤ 18 years, AIDS, pregnancy, organ or bone marrow transplant, immunosuppressive therapy including corticosteroids during the previous 30 days, and cancer or hematologic malignancy treated with radiotherapy or chemotherapy.

At inclusion, serum samples were collected to determine CMV serological status. Plasma samples were collected weekly for CMV PCR analysis. PCR results were reported, and antiviral therapy was prescribed according to the attending physician’s criteria.

Patients were followed up prospectively until death or hospital discharge.

### Definitions

**Major infections** included ventilator-associated pneumonia (VAP) or bacteremia.

VAP was defined as the presence of new and/or progressive pulmonary infiltrates on a chest radiograph in a patient ventilated for more than 48 hours plus 2 or more of the following criteria: fever ≥ 38.5°C or hypothermia ≤ 36°C, leukocytosis ≥ 12 × 10^9^ cells/L, purulent tracheobronchial secretions or a reduction in PaO2/FiO2 ≥ 15% in the previous 48 hours according to the definitions of the Centers for Disease Control and Prevention [24]. Patients with a clinical pulmonary infection score (CPIS) > 6 were also considered to have VAP [25]. The isolation of 1 or more microorganisms in a significant bacterial count was required to confirm the diagnosis.

An episode of **significant bacteremia** was defined as the presence of signs or symptoms of infection (fever ≥ 38.5°C or hypothermia < 36°C, and leukocytosis ≥ 12 × 10^9^ cells/L) and isolation of a bacterial or fungal pathogen in ≥ 1 blood cultures. We considered commensal microorganisms (coagulase-negative staphylococci, *Corynebacterium* species [except *C*. *jeikeium*], *Lactobacillus* species, *Bacillus* species, and *Propionibacterium* species, or viridans group streptococcus isolates, and *Clostridium perfringens*) as probable pathogens when they were recovered in ≥ 2 blood cultures (2 separate venipunctures).

### CMV assays

Antibodies to CMV were assessed using a commercial enzyme immunoassay kit for detection of total antibodies to CMV (LIAISON CMV IgG assay, DiaSorin S.p.A).

DNA was extracted in 200 μL of plasma eluted in 60 μL of elution buffer using a NucliSENS easyMAG system (bioMérieux, Boxtel, The Netherlands). A water sample was co-extracted as a negative control in all cases.

Samples were amplified using Affigene CMV Trender diagnostic assay (Cepheid AB, Bromma, Sweden), according to the manufacturer instructions. Amplification was performed on a MX3000P instrument (Stratagene Instruments Systems, La Jolla, CA, USA). Samples with >100 copies/mL of plasma were considered positive. Quantitative PCR levels were reported as copies per milliliter of plasma in samples with viral load >500 copies/ml.

Viral loads were obtained using the Affigene analysis software. Two calibrators of 2.6 × 10^3^ copies/ml and 3.6 ×10^8^ copies/ml were used to generate the standard curve. The quantitative range was between 500 copies/ml and 10^7^ copies/ml. The limit of detection was 88 copies/ml (95% confidence interval: 61–234 copies/ml). Samples with viral load between 100 copies/ml and 500 copies/ml were considered positive but viral load were not reported because the titre was below the range where precision for quantification had been determined.

### Statistical analysis

Qualitative variables appear with their frequency distribution. Normally distributed quantitative variables are expressed as the mean and standard deviation (SD); non-normally distributed quantitative variables are expressed as the median and interquartile range (IQR).

Normally distributed continuous variables were compared using the *t* test; non-normally distributed continuous variables were compared using the median test. The chi-squared or Fisher exact test was used to compare categorical variables. A p value < 0.05 was considered significant. All statistical tests were 2-tailed.

Logistic regression models were used to identify risk factors for CMV infection and for a composite endpoint of continued hospitalization or death by day 30. We also performed a landmark analysis of patients still hospitalized by 25 days after admission to the ICU in order to assess the probability of discharge for patients who had reactivated CMV and those who had not. We chose this time point because most patients had CMV reactivation by 25 days. Log-rank test were used to compare the hazards of discharge between groups.

Potential risk factors for CMV infection included; age, gender, history of diabetes, euroSCORE, transfused units mechanical, and ventilation at inclusion.

Potential risk factors for the composite endpoint included those mentioned above as well as CMV viremia and major infection.

Risk factors with a p value of <0.1 in the univariate analysis were entered into the multivariate model. The number of variables entered in the multivariate analysis was limited by the number of events (one variable for every 10 events). A p value <0.05 was considered significant.

All analyses were performed using SPSS V14 for Windows (SPSS Inc, Chicago, Illinois, USA) and STATA v.11.

## Results

### Study population

During the study period, 326 patients underwent MHS and 186/326 (57.1%) remained in the MHS-ICU for ≥ 3 days. Of 186 patients, 16 were excluded because of immunosuppression, 14 because they did not provide informed consent, and other reasons (n = 6). The remaining 150 patients constitute the study population. The characteristics of the study population are summarized in Table 1.

**Table 1:** Characteristics of the study population.

Characteristics	Study population (n = 150)
**Preoperative**	
Male gender, n (%)	88 (58.7)
Age (years), (mean ± SD)	67.9 ± 12.8
Underlying conditions, n (%)	
Myocardial infarction (<90 days)	26 (17.3)
Congestive heart failure	22 (14.7)
Cerebrovascular disease	23 (15.3)
Chronic obstructive pulmonary disease	26 (17.3)
Peripheral vascular disease	23 (15.3)
Gastrointestinal ulcer disease	18 (12)
Diabetes mellitus	37 (24.7)
Renal disease (creatinine Cr<2 mg/dL)	33 (22)
EuroSCORE, (mean ± SD)	8 (0–18)
Low risk (0–2), n (%)	13 (8.7)
Moderate risk (3–6), n (%)	42 (28)
High risk (>6), n (%)	95 (63.3)
**Operative**	
Surgical indication, n (%)	
Elective	122 (81.3)
Emergency	28 (18.7)
Type of surgery (%)	
Valve replacement	83 (55.3)
Coronary artery bypass grafting (CABG)	26 (17.3)
Mixed (valvular and CABG)	20 (13.3)
Aortic surgery	3 (2)
Other	17 (11.3)
**MHS ICU stay**	
Mechanical ventilation at inclusion	38 (25.3)
Vasoactive amines requirement, n (%)	83 (55.3)
Transfused patients, n (%)	130 (86.7)
Major infection, n (%)	33 (22)
Ventilator-associated pneumonia (VAP)	18
Bloodstream infection (BSI)	8
VAP + BSI	7
**Outcomes**	
Intensive care unit length of stay (d), median (range)	7 (3–165)
Hospital length of stay (d), median (range)	17 (4–352)
Mortality by day 30, n (%)	16 (10.7)
Hospitalized at day 30, n (%)	33 (22)

The primary composite endpoint of continued hospitalization or death by day 30 was recorded in 32.6% (49 of 150, 95% CI 25.3–40.7%) patients. There were no significant differences in the composite endpoint between seronegative (17 patients) and seropositive patients (35.3 vs. 32.3%, p = 0.8).

### Incidence of CMV infection

The seroprevalence of CMV in the study population was 88.7% (133 of 150, 95%CI 83.3–94%). Since all infected patients had positive CMV IgG at inclusion, we did not detect any cases of primary CMV infection.

CMV reactivation occurred in 16.5% (22 of 133 seropositive patients, CI 95% 9.2–20%) patients at a median of 17 days (range, 3–54 days) after MHS-ICU admission. Reactivation of CMV of >1000 copies/mL was recorded in 8.7% (13 of 150 patients included in the study, 95%CI 4–12.7%) at a median of 28 days (range, 21–56) after MHS-ICU admission.

Five patients received antiviral treatment with intravenous ganciclovir. No significant difference in the composite endpoint was observed between treated and untreated patients (100% vs 88.2%, p = 1).

### Risk factors for CMV infection

The univariate and multivariate analysis of risk factors for CMV infection are shown in Table 2. The multivariate model showed that diabetes (OR, 5.6; 95% CI, 1.8–17.4; p = 0.003) and high requirement for transfusions (OR, 13.7; 95% CI, 3.9–47.8; p<0.001) were independently associated with CMV infection.

**Table 2:** Univariate and multivariate analysis of risk factors for CMV infection.

Characteristics	Univariate analysis		Multivariate analysis	
	OR (95% CI)	p value	Adjusted OR	P value
Male gender, n (%)	1.3 (0.5–3.3)	0.6		
Age (10-year increments)	1.2 (0.8–1.7)	0.4		
Diabetes mellitus	3.9 (1.5–10)	0.004	5.6 (1.8–17.4)	0.003
EuroSCORE, (median, range)				
Low risk (0–2), n (%)	NA	NA		
Moderate risk (3–6), n (%)	0.7 (0.6–1.9)	0.7		
High risk (>6), n (%)	3.2 (4–16.4)	0.3		
Transfused units*				
0–5	NA	NA		
5–10	46 (5.6–375.4)	0.001		
>10	65.7 (7.8–553.6)	<0.001	13.7 (3.9–47.8)	P<0.001
Mechanical ventilation at inclusion	2.4 (0.9–6.1)	0.07	0.9 (0.3–3)	0.93

*red blood cells y/o platelets

### Risk factors for the composite endpoint

Table 3 shows the univariate and multivariate analysis of the factors associated with death or continued hospitalization by day 30 after MHS-ICU admission.

CMV viremia at any level was independently associated with hospitalization or death by day 30 (adjusted OR, 12.1; 95% CI, 2.3–64; p = 0.003). Patients who developed a major infection also had a higher risk of hospitalization or death by day 30 (Table 3).

**Table 3:** Risk factors for the composite end-point.

Characteristics	Univariate analysis		Multivariate analysis	
	OR (95% CI)	p value	Adjusted OR (95% CI)	p value
Male gender, n (%)	0.8(0.4–1.7)	0.7		
Age (10-year increments)	1.2 (0.9–1.6)	0.2		
EuroSCORE				
Low risk (0–2)	NA			
Moderate risk (3–6)	2.7 (0.3–24)	0.4		
High risk (>6),	9.9 (1.2–78.5)	0.003	3.8 (1.2–12)	0.02
Surgical indication, n (%)				
Emergency vs. elective	2.3 (0.7–7)	0.3		
Type of surgery (%)				
Valve replacement	1.7 (0.7–4.3)	0.2		
Coronary artery bypass grafting (CABG)	1.3 (0.4–3.6)	0.7		
Mixed (valve replacement and CABG)	1.4 (0.5–3.2)	0.7		
Aortic surgery	1.3 (0.4–3.9)	0.6		
Transfused units				
0–5	NA	NA		
5–10	11.5 (4.5–29.4)	<0.001		
>10	22.4 (7.3–68.4)	<0.001	4.1 (1.5–11.2)	0.005
Major infection, n (%)	9.8 (4.1–23.8)	<0.001	4.5 (1.4–14.9)	0.01
CMV viremia at any level	34.1 (7.5–154.7)	<0.001	12.1 (2.3–64)	0.003
Mechanical ventilation at inclusion	5.9 (2.6–13.2)	<0.001	0.4 (0.1–1.2)	0.10

Considering the possibility that longer length of stay (LOS) could lead to a greater possibility to detect CMV infection (spurious association), we studied CMV infection and hospital length of stay (LOS) in patients uniformly monitored for CMV reactivation. For this purpose, we performed a landmark analysis and assessed the cumulative incidence of time to discharge among the 48 patients who were still hospitalized by day 25 after admission. Patients were categorized as CMV infected if the PCR was positive prior to day 25. The median LOS after day 25 in CMV infected patients (n = 16) was 57 days (SD 9.2) compared with 78 days (SD 22.1) in non-infected (n = 32), p = 0.14).

## Discussion

The results of our study demonstrate that reactivation of CMV is a late complication that occurs frequently in immunocompetent MHS-ICU patients, particularly in diabetic patients with high transfusion requirements. CMV viremia was independently associated with continued hospitalization or death.

To our knowledge, only 2 previous studies [22, 23] have investigated CMV infection in patients undergoing heart surgery. Both studies were limited by their small sample size and the inclusion of selected patients. More recent studies analyzing reactivation of CMV in the ICU setting have included subsets of patients admitted to cardiac ICUs; however, the number of patients is too small for conclusions to be drawn [16, 17].

The incidence of CMV infection in immunocompetent ICU patients has been assessed in several studies [8–15] and reviewed in 2 recently published meta-analyses [16, 17]. If we focus on studies in which PCR assay was used for monitoring at least weekly [14, 26], as in our study, the rate of CMV infection was 32% to 33%. We found that CMV reactivated in 16.5% of the immunocompetent patients who underwent MHS. This incidence is lower than that mentioned above, although it is consistent with that reported in studies involving small subsets of MHS-ICU patients [14].

Several risk factors for reactivation of CMV in immunocompetent ICU patients have been reported [16, 17]. We found that patients with diabetes mellitus and high transfusions requirement (>10 units) were at increased risk of CMV infection (adjusted OR, 5.6 and 13.7, respectively). Diabetic patients have an impaired immune response that makes them more susceptible to infection. Moreover, since CMV seroprevalence has been reported to be higher in diabetic patients [27, 28], it seems reasonable to assume that diabetic patients are at higher risk of CMV reactivation.

Regarding transfusions, several hypotheses may explain its association with CMV infection. Nowadays, with the use of leukocyte depleted products, the risk or transfusion-transmitted CMV infection is low [29]. However, some studies have suggested that after leukoreduction post-transfusion CMV infection in seronegative patients, while uncommon, remains possible [30]. In our study, we did not observe any CMV infection in seronegative patients. As for seropositive patients, CMV reinfection with an exogenous strain could occur but this fact is very difficult to demonstrate. We think that the most plausible explanation for the association between multiple transfusions and CMV reactivation may be the effect of postoperative bleeding on the immunomodulatory response [31].

In the present study, we found that reactivation of CMV was associated with hospitalization or death by day 30 in patients undergoing MHS. However, due to the observational study design, we could not demonstrate causality. We could not confirm the association of longer duration of subsequent hospitalization in the subset of patients who remained in the hospital after 25 days, although there was a trend towards longer hospitalization or death in patients with CMV infection.

Remarkably, we also found that the disease severity (assessed using the euroSCORE) was not associated with an increased risk of developing CMV infection. Thus, reactivation of CMV does not appear to be simply a marker for disease severity in this setting. The mechanisms leading to an association between reactivation and adverse outcomes are unknown. One plausible explanation for the consequences of reactivation may be its indirect immunomodulatory effects [32]. Other biological mechanisms, such as, direct lung injury, amplification of inflammation signals, or predisposition to nosocomial infections have been suggested to link CMV reactivation with adverse outcomes [33]. However, we believe that a causal association between these effects can only be assessed by means of a randomized controlled trial of CMV treatment in this setting. Fortunately, a randomized controlled trial of ganciclovir in ICU patients is underway (NCT01335932).

The strengths of the present study include its prospective design; large and homogenous study population, use of a quantitative CMV PCR assay, and robust statistical analyses.

Nevertheless, the study is also subject to limitations. 1) although currently it is recommend to report the viral load as international units (IU), at the time of the study we were not able to convert copies/ml to IU because the CMV assay used were not calibrate with the WHO CMV International standard, 2) the administration of antiviral treatment based on clinical judgment might have modified the natural history of the disease, however we did not find significant difference in the composite endpoint between treated and untreated patients (100% vs 88.2%, p = 1), 3) given its observational design, we cannot infer causality between reactivation of CMV and the composite endpoint. Future clinical trials are needed to clarify whether CMV is a real pathogen in this population and evaluate whether prevention and/or treatment of CMV reactivation will improve clinical outcome.

In conclusion, reactivation of CMV is frequent in immunocompetent patients undergoing MHS and appears to contribute to worse clinical outcome.

## References

[1] De Vlieger G, Meersseman W, Lagrou K, Wouters P, Wilmer A, Peetermans WE, et al Cytomegalovirus serostatus and outcome in nonimmunocompromised critically ill patients. Critical care medicine. 2012;40(1):36–42. Epub 2011/09/20. 10.1097/CCM.0b013e31822b50ae . 21926616

[2] Blyth D, Lee I, Sims KD, Gasink LB, Barton TD, Van Deerlin VM, et al Risk factors and clinical outcomes of cytomegalovirus disease occurring more than one year post solid organ transplantation. Transplant infectious disease: an official journal of the Transplantation Society. 2012;14(2):149–55. Epub 2012/01/21. 10.1111/j.1399-3062.2011.00705.x . 22260410

[3] Harvala H, Stewart C, Muller K, Burns S, Marson L, MacGilchrist A, et al High risk of cytomegalovirus infection following solid organ transplantation despite prophylactic therapy. Journal of medical virology. 2013;85(5):893–8. Epub 2013/03/20. 10.1002/jmv.23539 . 23508914

[4] Kijpittayarit-Arthurs S, Eid AJ, Kremers WK, Pedersen RA, Dierkhising RA, Patel R, et al Clinical features and outcomes of delayed-onset primary cytomegalovirus disease in cardiac transplant recipients. The Journal of heart and lung transplantation: the official publication of the International Society for Heart Transplantation. 2007;26(10):1019–24. Epub 2007/10/09. 10.1016/j.healun.2007.07.016 . 17919622

[5] Linares L, Sanclemente G, Cervera C, Hoyo I, Cofan F, Ricart MJ, et al Influence of cytomegalovirus disease in outcome of solid organ transplant patients. Transplantation proceedings. 2011;43(6):2145–8. Epub 2011/08/16. 10.1016/j.transproceed.2011.05.007 . 21839217

[6] Benhammane H, Mentha G, Tschanz E, El Mesbahi O, Dietrich PY. Visceral Kaposi's Sarcoma Related to Human Herpesvirus-8 in Liver Transplant Recipient: Case Report and Literature Review. Case reports in oncological medicine. 2012;2012:137291 Epub 2013/01/16. 10.1155/2012/137291 23320218 PMC3539345

[7] Snyder LD, Finlen-Copeland CA, Turbyfill WJ, Howell D, Willner DA, Palmer SM. Cytomegalovirus pneumonitis is a risk for bronchiolitis obliterans syndrome in lung transplantation. American journal of respiratory and critical care medicine. 2010;181(12):1391–6. Epub 2010/02/20. 10.1164/rccm.200911-1786OC 20167845 PMC2894412

[8] Stephan F, Meharzi D, Ricci S, Fajac A, Clergue F, Bernaudin JF. Evaluation by polymerase chain reaction of cytomegalovirus reactivation in intensive care patients under mechanical ventilation. Intensive care medicine. 1996;22(11):1244–9. Epub 1996/11/01. . 9120120 10.1007/BF01709343 PMC7094969

[9] Cook CH, Martin LC, Yenchar JK, Lahm MC, McGuinness B, Davies EA, et al Occult herpes family viral infections are endemic in critically ill surgical patients. Critical care medicine. 2003;31(7):1923–9. Epub 2003/07/09. 10.1097/01.ccm.0000070222.11325.c4 . 12847384

[10] Cook CH, Yenchar JK, Kraner TO, Davies EA, Ferguson RM. Occult herpes family viruses may increase mortality in critically ill surgical patients. American journal of surgery. 1998;176(4):357–60. Epub 1998/11/17. . 9817255 10.1016/s0002-9610(98)00205-0

[11] Heininger A, Haeberle H, Fischer I, Beck R, Riessen R, Rohde F, et al Cytomegalovirus reactivation and associated outcome of critically ill patients with severe sepsis. Critical care (London, England). 2011;15(2):R77 Epub 2011/03/03. 10.1186/cc10069 21362193 PMC3219329

[12] Heininger A, Jahn G, Engel C, Notheisen T, Unertl K, Hamprecht K. Human cytomegalovirus infections in nonimmunosuppressed critically ill patients. Critical care medicine. 2001;29(3):541–7. Epub 2001/05/25. . 11373417 10.1097/00003246-200103000-00012

[13] Jaber S, Chanques G, Borry J, Souche B, Verdier R, Perrigault PF, et al Cytomegalovirus infection in critically ill patients: associated factors and consequences. Chest. 2005;127(1):233–41. Epub 2005/01/18. 10.1378/chest.127.1.233 . 15653989

[14] Limaye AP, Kirby KA, Rubenfeld GD, Leisenring WM, Bulger EM, Neff MJ, et al Cytomegalovirus reactivation in critically ill immunocompetent patients. JAMA: the journal of the American Medical Association. 2008;300(4):413–22. Epub 2008/07/24. 10.1001/jama.300.4.413 18647984 PMC2774501

[15] Ziemann M, Sedemund-Adib B, Reiland P, Schmucker P, Hennig H. Increased mortality in long-term intensive care patients with active cytomegalovirus infection. Critical care medicine. 2008;36(12):3145–50. Epub 2008/10/22. 10.1097/CCM.0b013e31818f3fc4 . 18936696

[16] Osawa R, Singh N. Cytomegalovirus infection in critically ill patients: a systematic review. Critical care (London, England). 2009;13(3):R68 Epub 2009/05/16. 10.1186/cc7875 19442306 PMC2717427

[17] Kalil AC, Florescu DF. Prevalence and mortality associated with cytomegalovirus infection in nonimmunosuppressed patients in the intensive care unit. Critical care medicine. 2009;37(8):2350–8. Epub 2009/06/18. 10.1097/CCM.0b013e3181a3aa43 . 19531944

[18] Kollef MH. Ventilator-associated pneumonia. A multivariate analysis. JAMA: the journal of the American Medical Association. 1993;270(16):1965–70. Epub 1993/10/27. . 8411554

[19] Kollef MH, Sharpless L, Vlasnik J, Pasque C, Murphy D, Fraser VJ. The impact of nosocomial infections on patient outcomes following cardiac surgery. Chest. 1997;112(3):666–75. Epub 1997/10/07. . 9315799 10.1378/chest.112.3.666

[20] Sodano L, Agodi A, Barchitta M, Musumeci F, Menichetti A, Bellocchi P, et al Nosocomial infections in heart surgery patients: active surveillance in two Italian hospitals. Annali di igiene: medicina preventiva e di comunita. 2004;16(6):735–43. Epub 2005/02/09. . 15697003

[21] Bouza E, Hortal J, Munoz P, Pascau J, Perez MJ, Hiesmayr M. Postoperative infections after major heart surgery and prevention of ventilator-associated pneumonia: a one-day European prevalence study (ESGNI-008). The Journal of hospital infection. 2006;64(3):224–30. Epub 2006/08/26. 10.1016/j.jhin.2006.06.019 . 16930769

[22] Domart Y, Trouillet JL, Fagon JY, Chastre J, Brun-Vezinet F, Gibert C. Incidence and morbidity of cytomegaloviral infection in patients with mediastinitis following cardiac surgery. Chest. 1990;97(1):18–22. Epub 1990/01/01. . 2153065 10.1378/chest.97.1.18

[23] Caul EO, Mott MG, Clarke SK, Perham TG, Wilson RS. Cytomegalovirus infections after open heart surgery. A prospective study. Lancet. 1971;1(7703):777–80. Epub 1971/04/17. . 4101275 10.1016/s0140-6736(71)91216-5

[24] Garner JS, Jarvis WR, Emori TG, Horan TC, Hughes JM. CDC definitions for nosocomial infections, 1988. American journal of infection control. 1988;16(3):128–40. Epub 1988/06/01. . 2841893 10.1016/0196-6553(88)90053-3

[25] Pugin J, Auckenthaler R, Mili N, Janssens JP, Lew PD, Suter PM. Diagnosis of ventilator-associated pneumonia by bacteriologic analysis of bronchoscopic and nonbronchoscopic "blind" bronchoalveolar lavage fluid. The American review of respiratory disease. 1991;143(5 Pt 1):1121–9. Epub 1991/05/01. 10.1164/ajrccm/143.5_Pt_1.1121 . 2024824

[26] Kutza AS, Muhl E, Hackstein H, Kirchner H, Bein G. High incidence of active cytomegalovirus infection among septic patients. Clinical infectious diseases: an official publication of the Infectious Diseases Society of America. 1998;26(5):1076–82. Epub 1998/05/23. . 9597229 10.1086/520307

[27] Visseren FL, Bouter KP, Pon MJ, Hoekstra JB, Erkelens DW, Diepersloot RJ. Patients with diabetes mellitus and atherosclerosis; a role for cytomegalovirus? Diabetes Res Clin Pract. 1997;36(1):49–55. 9187415 10.1016/s0168-8227(97)00027-2

[28] Roberts BW, Cech I. Association of type 2 diabetes mellitus and seroprevalence for cytomegalovirus. Southern medical journal. 2005;98(7):686–92. 16108236 10.1097/01.SMJ.0000163310.12516.2D

[29] Cervia JS, Wenz B, Ortolano GA. Leukocyte reduction's role in the attenuation of infection risks among transfusion recipients. Clinical infectious diseases: an official publication of the Infectious Diseases Society of America. 2007;45(8):1008–13. Epub 2007/09/21. 10.1086/521896 . 17879916

[30] Ziemann M, Hennig H. Prevention of Transfusion-Transmitted Cytomegalovirus Infections: Which is the Optimal Strategy? Transfus Med Hemother. 2014;41(1):40–4. Epub 2014/03/25. 10.1159/000357102 ; PubMed Central PMCID: PMCPmc3949610. 24659946 PMC3949610

[31] Angele MK, Faist E. Clinical review: immunodepression in the surgical patient and increased susceptibility to infection. Critical care (London, England). 2002;6(4):298–305. Epub 2002/09/13. ; PubMed Central PMCID: PMCPmc137309. 12225603 10.1186/cc1514 PMC137309

[32] Boeckh M, Nichols WG. Immunosuppressive effects of beta-herpesviruses. Herpes: the journal of the IHMF. 2003;10(1):12–6. Epub 2003/05/17. . 12749798

[33] Limaye AP, Boeckh M. CMV in critically ill patients: pathogen or bystander? Reviews in medical virology. 2010;20(6):372–9. Epub 2010/10/12. 10.1002/rmv.664 20931610 PMC2987685

